# The Effects of 24-h Fasting on Exercise Performance and Metabolic Parameters in a Pilot Study of Female CrossFit Athletes

**DOI:** 10.3390/nu15224841

**Published:** 2023-11-20

**Authors:** Melike Nur Eroglu, Celia Rodríguez-Longobardo, Ana Ramírez-Adrados, Clara Colina-Coca, Silvia Burgos-Postigo, Olga López-Torres, Valentín E. Fernández-Elías

**Affiliations:** 1 Coaching Education Department, Sports Science Faculty, Sakarya University of Applied Sciences, Serdivan 54050, Turkey; melikeeroglu@subu.edu.tr; 2 Social Sciences of Physical Activity, Sport and Leisure Department, Faculty of Physical Activity and Sport Sciences, Universidad Politécnica de Madrid, 28040 Madrid, Spain; celia.rlongobardo@upm.es; 3 Faculty of Sports Sciences, Universidad Europea de Madrid, 28670 Villaviciosa de Odón, Spain; ana.ramirez@universidadeuropea.es (A.R.-A.); silvia.burgos@universidadeuropea.es (S.B.-P.); valentin.fernandez@universidadeuropea.es (V.E.F.-E.); 4 Faculty of Biomedical and Health Sciences, Universidad Europea de Madrid, 28670 Villaviciosa de Odón, Spain; clara.colina@universidadeuropea.es

**Keywords:** intermittent fasting, crossFit, exercise performance, fatigue, blood lactate

## Abstract

Many studies have tested intermittent fasting (IF) in athletes, but its effects on female CrossFit athletes remain relatively unexplored in the existing literature. The aim of this study was to evaluate and compare the effects of 24-h IF on the physical performance of female CrossFit practitioners. Eleven female CrossFit athletes (age: 30.91 ± 3.42, weight: 65.26 ± 7.55 kg, height: 1.66 ± 0.05 m) participated in the study. The study used a crossover design with fasting and eating conditions. Participants completed an exercise test, standing long jump, and handgrip strength assessment. Hydration status, heart rate, blood lactate, blood glucose, rates of perceived exertion, and hunger were measured. Results showed significant differences in blood lactate concentration (F = 5.435, *p* = 0.042, η2p = 0.352). Resting blood lactate concentration was significantly lower in the fasting trial than in the eating trial (*p* < 0.001), but post-exercise blood lactate concentrations were higher in the fasting trial than in the eating trial (*p* < 0.001). No differences were found in performance times (*p* > 0.05). In conclusion, this pilot study of females suggests that 24-h fasting does not impair exercise performance or negatively affect physiological parameters in CrossFit athletes.

## 1. Introduction

Intermittent fasting (IF) is a popular dietary practice, which consists of regular alternating periods of unrestricted dietary consumption and abstinence from caloric intake. In recent years, various forms of IF have been extensively investigated with the aim of improving exercise performance and enhancing metabolic health.

There have been several proposed protocols/methods of IF that are in practice. Some of the common ones include alternate-day fasting, which involves fasting for 24-h every other day, and the 5:2 method, which involves a fast of 24-h twice a week and a very low-calorie diet consumed two other days of the week. In these protocols, fasting can take place on consecutive or non-consecutive days [1]. Fasting is also an important part of many religions, a notable example being Muslims fasting during Ramadan, a month-long period during which no food or liquid is consumed during daylight hours [2].

These fasting protocols have been associated with various health benefits such as increasing metabolic flexibility, improving insulin sensitivity, promoting weight loss, and even slowing down the aging process. However, our knowledge about the effects of IF modalities as a factor influencing athletic performance is limited.

Specifically, short-term (acute) fasting protocols such as the 24-h fasting period can impact exercise performance. During this period, the body utilizes glycogen stores and increases fat metabolism to provide energy. This can lead to changes in energy production mechanisms and potentially affect exercise performance positively or negatively [3].

The effects of short-term fasting on exercise performance have been extensively investigated, and suggest that decreased physical performance occurs during the fasting state [4]. This could be explained (at least in part) by the fasting periods used (>24 to 55 h), dehydration, prolonged exhaustive exercise tests [5,6,7], and/or very high-intensity levels of exercise [7,8]. However, others [9,10,11,12] failed to record significant decreases in performance after shorter periods of fasting (11–24 h).

CrossFit is a high-intensity fitness training program that combines elements of weightlifting, cardiovascular exercise, and bodyweight movements. It aims to improve overall fitness by focusing on functional movements performed at a high intensity. CrossFit workouts, known as “WOD” (Workout of the Day), are constantly varied and incorporate exercises from various disciplines, such as weightlifting, gymnastics, and metabolic conditioning [13].

Different nutritional strategies and approaches are used to improve the performance of CrossFit practitioners, and intermittent fasting can be compatible with CrossFit as individuals can schedule their eating windows around their workouts. Aerobic and anaerobic energy production processes form the basis of CrossFit training, and how these energy systems are affected under IF diets is not yet fully understood since most of them are empirical and lack scientific evidence [14,15]. Studies have focused more on selected nutritional interventions, such as the ketogenic diet [16,17], pre- and post-workout protein, and carbohydrate intake [18,19,20,21].

In a study of 2576 CrossFit practitioners, it was reported that 7.7% of the participants were on an IF diet [22]. Considering sex when prescribing dietary strategies is important because men and women have distinct physiological differences (hormonal variations, body composition, and metabolic rates) that can affect their nutritional needs and health outcomes [23]. A recent study [24] surveyed 449 CrossFit practitioners about their workouts and dietary intake and concluded that it is necessary to consider sex when prescribing dietary strategies. Specifically, it has been reported that women more often choose weight loss and fat loss as their nutritional goals, while men are more likely to choose to gain muscle mass and weight. These findings are consistent with previous research on exercise behavior where women exercise more frequently than men to lose weight and men tend to gain muscle mass/weight [25,26].

Based on the information presented, the effects of IF diets on female CrossFit athletes remain relatively unexplored in the existing literature. Therefore, the aim of this study was to assess and compare the effects of 24 h fasting on the physical performance of female CrossFit practitioners. Our hypothesis was that a 24 h fasting period would not negatively affect physical performance compared with a normal pre-exercise diet.

## 2. Material and Methods

### 2.1. Participant Selection and Study Design

Eleven female crossfitters (characteristics: age: 30.91 ± 3.42, weight: 65.26 ± 7.55 kg, height: 1.66 ± 0.05 m) participated in the study. The number of participants was determined according to the power analysis results. All were recruited from the same local CrossFit gym. Recruitment and study took place in July 2023. Inclusion criteria were: (1) being female; (2) being 18 or older; and (3) having CrossFit training experience for at least 2 years. The exclusion criteria were: (1) following pharmacological treatment or supplement (including the use of stimulants such as caffeine), (2) muscular, ligamentous, bone, nerve, or joint pathology incompatible with the training program; (3) present cardiovascular or cardiorespiratory problems; and (4) performance of other sports activities during their participation in the study that could influence the study results. All participants were assessed in the same menstrual cycle phase (late follicular, after the subject’s reported menstruation) to avoid the effects of the menstrual cycle phase on exercise capacity [27].

Before commencing the study, all subjects were informed about the experimental procedures and the possible risks and benefits of the study. Each subject signed a written informed consent form prior to participation. The study protocol was approved by the Local Clinical Research Ethics Committee of Madrid (47/764390/17) and was in accordance with the Declaration of Helsinki.

The present study used a within-subject counterbalanced crossover design, as this design has many advantages: each subject acted as their own control, thus reducing possible error variance, while at the same time reducing the required sample size. Each participant was tested under fasting and non-fasting conditions. While the fasting + exercise trial was conducted in the first week, a normal diet + exercise trial was conducted on the same day and time one week later. Participants completed an exercise test, a standing long jump, and a handgrip strength test. Hydration status, heart rate, blood lactate, blood glucose, perceived exertion, and hunger were measured. All participants performed the same set of tests in the same order on each trial. To eliminate the learning effect, the protocols were explained to the participants in advance and practiced. They were asked to refrain from any strenuous exercise 24 h before the test.

### 2.2. Nutrition Protocol

The participants were provided with the fasting/eating protocol prior to each test period. It was therefore not possible to blind study participants to the testing conditions. In the fasting trial, participants consumed only water for 24 h before exercise. In the eating trial, the instruction given to the participants was to have their last meal previous to the assessment within 2–3 h prior to exercise. On the other hand, participants were provided with nutritional guidelines for the meal before the physical test, which consisted of consuming a meal composed of 50% carbohydrates, 25% fats, and 25% proteins according to the validated visual nutritional tool Athlete’s Plate^®^ [28].

With the aim of promoting adherence, all subjects were briefed on the hard training day Athlete’s Plate^®^ protocol, as it adjusts the composition of main food groups on the plate to align with high-intensity training days and meet international sports nutrition guidelines [29,30]. Furthermore, a categorized list of foods based on their primary macronutrient content was provided, along with meal ideas, to empower participants to make well-informed dietary decisions.

The athletes were encouraged to contact the dieticians with any questions or concerns regarding the nutritional protocol. All instructions about nutritional guidelines were provided by registered dieticians.

In both test conditions, participants were asked to avoid strenuous physical activity to minimize the potential carry-over effects such as fatigue, muscle damage, or physiological stress. Prior to each testing period, a pre-trial checklist was completed to collect information about the participants’ activities over the previous 24-h (e.g., time of last meal, exercise performed, and any injuries since the last contact).

### 2.3. Physical Testing

#### 2.3.1. Exercise Test

The exercise test consisted of 5 sets of 10 × burpee over bar, 10 × pendlay rowing, 10 × deep squats, and 4 × 20 m sprints. Participants were asked to be as fast as possible, and times were recorded the assess physical performance.

#### 2.3.2. Standing Long Jump

The standing long jump test was used to measure strength and sprint speed [31]. Participants were instructed to perform a standing long jump from a position behind a designated starting line, with the aim of achieving the maximum distance forward using both legs. The distance covered during the jump was quantified in centimeters, determined by measuring from the front edge of the starting line to the point of initial heel contact with the ground.

#### 2.3.3. Handgrip Strength

Handgrip strength test was performed as an indicator of overall strength [32]. This test was performed using a hydraulic hand-held dynamometer (Takei 5001, Japan) with an accuracy of 0.1 kg. Before and after the test, the subjects held a standardized position (standing, with the elbow in full extension) for 2–3 s at maximum pressure. All participants repeated the test twice, alternating between each hand. The researchers recorded the best score from the two trials.

### 2.4. Measurements

#### 2.4.1. Hydration Level

Immediately before exercise, the participants were asked to provide a urine sample. A standard well-established urine color scale [33], with demonstrated test-retest reliability and validity [34,35], was used to assess hydration status. The urine samples were held against a white background and the urine color scores were determined using numbers to describe the hydration status, i.e., numbers 1–3 indicate hyperhydrated hydration status, 4 indicate euhyrated hydration status, and 5–8 indicate hypohydrated hydration status.

#### 2.4.2. Heart Rate

The heart rate was monitored during exercise and at rest using a Polar H10 heart rate monitor (Kempele, Finland). The Elite HRV (Heart Rate Variability, NC, ABD) application was used as the data receiver in a Bluetooth connection to record HR (HR average) and measure heart rate variability (HRV). The Elite HRV application has been previously validated for HRV recording [36]. For HRV measurement, the square root of the mean of the sum of the squared differences between normal adjacent RR intervals (RMSSD), the low-frequency band (LF), and the high-frequency band (HF) were analyzed [37].

#### 2.4.3. Blood Lactate

Using a disposable lancet device, the skin was punctured just at the center of the finger pad. The first drop of blood was wiped away, and then approximately 5 μL (2 mm) of blood was applied to the lactate strip and immediately analyzed using the Lactate Pro Analyser (ARKRAY Inc., Kyoto, Japan) [38]. Blood lactate concentrations were measured at rest (pre-exercise) and immediately after the completion of the exercise protocol (post-exercise).

#### 2.4.4. Blood Glucose

Blood glucose measurements were taken from the fingertip using a glucose analyzer (Freestyle Optium Neo, Abbot, Madrid, Spain). The blood glucose was measured both before and immediately after the completion of the 5 sets performed by the participants. Blood sampling was carried out immediately after lactate analysis.

#### 2.4.5. Rate of Perceived Exertion (RPE)

The Borg CR-10 scale was used to assess the perceived exertion. The assessments were made pre- and post-exercise. Participants were given detailed instructions on how to rate the experience in terms of perceived exertion. Each participant rated the perception of physical effort on a scale from “absolutely nothing” (score 0) to “extremely strong” (score 10) [39].

#### 2.4.6. Hunger Feelings

Hunger feeling was assessed pre- and post-training using a Visual Analog Scale (VAS), where participants had to rate their sensation from 0 to 10. A score of 0 represents the “absence of hunger sensation” and 10 represents “very hungry”. The VAS method has been validated in several populations [40].

### 2.5. Statistical Analysis

Statistical analysis was performed with the Statistical Package for the Social Sciences (SPSS, version 24, SPSS Inc., Chicago, IL, USA) in the Windows environment. Data are presented as mean ± SD. The Kolmogorov–Smirnov test was used to confirm a normal distribution of data. To assess variables measured pre- and post-exercise, a two-way analysis of variance (ANOVA) with repeated measures was conducted to determine the main effects of the experimental trials (fasting vs. pre-competition diet) and time (pre- vs. post-exercise) as well as interaction (experimental trial × time). Bonferroni post hoc analysis was performed when a significant F value (Greenhouse–Geisser adjustment for sphericity) was observed. For single measure variables, a Student *t*-test was performed. The effect size was estimated by calculating partial eta squared (η2p) and Cohen’s d, for pre- and post-exercise and single measure variables, respectively.

## 3. Results

**Figure 1:**
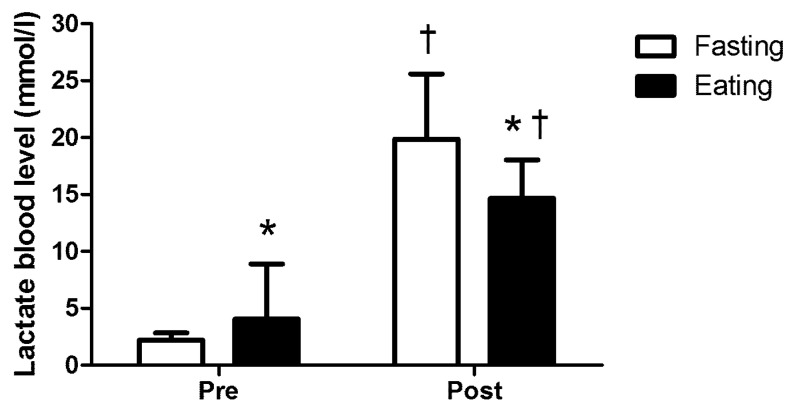
Pre- and post-exercise lactate blood levels. * Significantly different than fasting trial (*p* < 0.05). † Significantly different than pre-exercise (*p* < 0.05).

The ANOVA analysis revealed significant differences in blood lactate concentration (F = 5.435, *p* = 0.042, η2p = 0.352). The resting blood lactate concentration in the fasting trial was significantly lower than that in the eating trial (*p* < 0.001). Interestingly, post-exercise blood lactate concentrations were higher in the fasting trial compared to the eating trial (*p* <0.001, Figure 1). There was no significant difference between trials in RPE (*p* > 0.05, Table 1).

**Figure 2:**
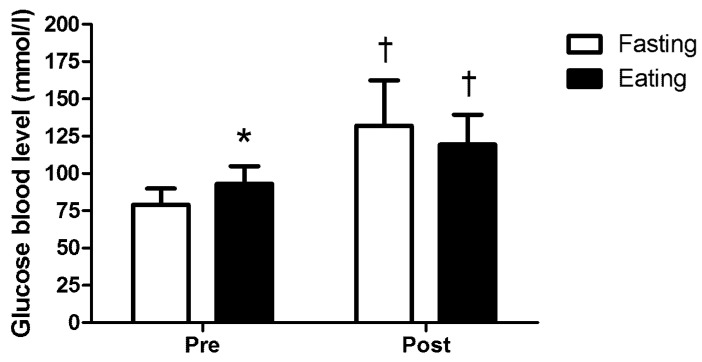
Pre- and post-exercise glucose blood levels. * Significantly different than fasting trial (*p* < 0.05). † Significantly different than pre-exercise (*p* < 0.05).

In terms of blood glucose concentration (F = 16.80, *p* = 0.002, η2p = 0.627), resting levels were significantly lower in the fasting trial than in the eating trial (*p* < 0.001). However, the increase in blood glucose concentration from pre- to post-exercise was not significantly different between the two conditions (Figure 2).

Participants reported higher levels of hunger both pre- and post-exercise in the fasting trial compared to the eating trial (*p* < 0.001, Table 1).

Handgrip strength values measured with both the right and left hand were not affected by the fasting or eating trials. No significant differences were observed pre- and post-exercise (*p* > 0.05). In addition, there were no significant differences in standing jumping distances between pre- and post-exercise measurements in either the fasting or eating trials (*p* > 0.05, Table 1).

No significant differences in performance times were found between the fasting and eating trials (*p* > 0.05). Resting heart rates before exercise were not significantly different between the fasting and eating trials (71.3 ± 8.3 vs. 67.42 ± 7.44 bpm, fasting vs. eating, respectively, *p* = 0.131, t = 1.647, η2 = 0.49). Additionally, heart rate values (RMSSD, LF, HF, Mean HR) during exercise were similar between the fasting and eating trials (Table 2).

Finally, no significant difference in pre-exercise hydration levels was observed between the fasting and eating trials (2.33 ± 1.33 vs. 2.1 ± 0.87, fasting vs. eating, respectively, *p* = 0.662, t = 0.452, η2 = 0.17).

## 4. Discussion

Our study aimed to investigate the effects of fasting on exercise performance and related physiological parameters in a group of female CrossFit athletes. The results showed that 24-h fasting can lead to lower resting blood lactate concentrations, increased post-exercise lactate levels, and higher subjective hunger levels. However, fasting did not significantly affect ratings of perceived exertion, post-exercise blood glucose concentration, handgrip strength, jumping performance, performance times, heart rate parameters, or hydration levels.

Blood lactate concentration is an important indicator of the metabolic response to exercise and fatigue levels. Our results showed that resting blood lactate levels were significantly lower in the fasting trial compared to the eating trial. The lower resting lactate levels observed in the fasting trial could be attributed to a decrease in carbohydrate availability, leading to reduced resting glycolytic activity and thus lower lactate production.

Post-exercise blood lactate concentrations were higher in the fasting trial compared to the eating trial. This indicates that fasting may lead to increased lactate production during exercise. Previous research on athletes has shown that fasting can improve lactate production and clearance during exercise [12,41]. The higher post-exercise lactate levels observed in the fasting trial could be attributed to an increased reliance on glycogenolysis and subsequent lactate production as an alternative energy source in the absence of readily available carbohydrates. This increased lactate production may have resulted from the recruitment of a greater mass of higher threshold glycolytic fast-twitch fibers, as the population of slow-twitch fibers became increasingly glycogen-depleted and fatigued. Loy et al. [7] found a significant increase in lactate concentration accompanied by a decrease in skeletal muscle glycogen after a 24-h fasting and exercise to exhaustion. Furthermore, elevated norepinephrine levels in the fasting trial may have impeded lactate clearance by diverting blood flow away from the liver [7,42].

Despite the differences in blood lactate concentrations, we did not observe any significant differences in RPE between the fasting and eating trials. This suggests that subjective perceptions of effort during exercise were similar regardless of the fasting state. Similar findings have been reported in previous studies comparing RPE between fasting and eating states [43]. It is possible that other factors, such as motivational factors or the participants’ adaptation to training in a fasted state, may have influenced their perceived exertion levels [44].

Our study also investigated the effects of fasting on blood glucose concentration. Resting blood glucose levels were significantly lower in the fasting trial compared to the eating trial, which is consistent with previous research showing reduced blood glucose levels during fasting [12,45,46]. However, the increase in blood glucose concentration from pre- to post-exercise was not significantly different between the two conditions. This suggests that the body’s compensatory mechanisms, such as increased gluconeogenesis and glycogen breakdown, adequately maintain blood glucose levels during exercise regardless of the fasting state [4].

The fasting trial had higher hunger levels both pre- and post-exercise compared to the eating trial. The subjective experience of hunger was influenced by hormonal changes and lack of food intake, which can lead to higher perceived hunger levels in fasting individuals. The feeling of hunger post-exercise decreased in both trials. This effect is due to the ability of acute exercise to temporarily suppress appetite-regulating hormones [47].

A recent meta-analysis of twenty-eight studies suggests that IF does not affect muscle strength and anaerobic capacity [48]. In terms of physical performance, fasting did not significantly affect performance time, handgrip strength values, or standing jumping distances in our study. The effect of short-term fasting on exercise capacity has been extensively investigated, with many studies concluding that fasting prior to exercise results in impaired performance [8,12]. This outcome has been attributed, in part, to the adoption of prolonged fasting periods (>24 to 55 h), dehydration [10], prolonged exhaustive exercise testing [5,6], or engaging in very high-intensity levels of exercise [7,8]. However, some researchers have failed to observe a significant decline in performance after shorter fasting periods (11–24 h) [10,12]. This discrepancy in findings could be potentially explained by the glycogen-sparing effect of fasting before exercise, which is linked to increased availability of free fatty acids, thus accounting for the absence of performance decline during short-term fasting [9,11].

Our study showed that performance time exhibits a nonsignificant trend, being 7% longer after fasting compared to the eating state. As we acclimatized the participants to the exercise protocol, we do not believe that the difference could be due to a learning effect. However, this could be due to the small sample size, which is a limitation of the present study. In this line, the β level for performance analysis was 0.996, which indicates that the statistical power was low. Thus, the null hypothesis may not be rejected.

Resting heart rate and heart rate variability during exercise were not significantly different between the fasting and eating trials. There is conflicting information about the effect of fasting on heart rate during exercise. Sabah Hammoud et al. [49] found that fasting during Ramadan significantly increased HRV in the afternoon. Mzurak et al. [50] confirm that an acute (48 h) total fasting induces parasympathetic withdrawal with simultaneous sympathetic activation. These changes appear to reflect stress, which may be related to feelings of hunger. The most likely explanation of the effect of attenuation of HR in the fasting state compared to the eating state may be related to a higher parasympathetic activity during fasting [51,52]. The variations in these findings may be attributed to differences in fasting duration, exercise intensity, and participant characteristics. Future studies with larger sample sizes and more comprehensive monitoring of heart rate parameters are warranted to provide a clearer understanding of the relationship between fasting and cardiovascular responses to exercise.

Finally, our study found no significant difference in hydration levels between the fasting and eating trials. This suggests that participants maintained adequate hydration status in both conditions. Hydration is crucial for optimal exercise performance. However, as far as we know, no study has investigated the effects of 24-h fasting on hydration status. However, previous research [48] has reported that prolonged fasting has a detrimental effect on hydration status and impairs performance.

The present study is not without limitations. As mentioned earlier, the sample size was small, and a larger number of participants could have yielded different statistical outcomes. Additionally, another limitation is the absence of additional parameters that could be relevant in a fasting-exercise context, such as blood fatty acids and levels of stress-related hormones like cortisol.

## 5. Conclusions

In conclusion, these female-specific findings in a small pilot study are generally consistent with previous research on athletes and support the concept that short-term fasting does not impair exercise performance or negatively impact physiological parameters in female CrossFit athletes. Future research with larger sample sizes and diverse athlete populations is needed to further validate and extend these findings.

## Figures and Tables

**Table 1:** Pre- and post-exercise measurements.

		Pre-Exercise	Post-Exercise	F	*p*	η2p	Post Hoc
RPE (1;10)	Fasting	4.09 ± 2.02	8.18 ± 1.32	0.009	0.928	0.001	
	Eating	3.09 ± 1.51	7.27 ± 0.78				
Ratings of Hunger (0;10)	Fasting	5.86 ± 3.03	1.90 ± 1.97	8.213	0.017	0.451	Pre-fasting vs. eating *p* < 0.001: Post-fasting vs. eating *p* < 0.001
	Eating	1.54 ± 1.91	0.63 ± 1.56				
HGS Right (kg)	Fasting	32.95 ± 3.61	33.68 ± 2.76	0.084	0.777	0.008	
	Eating	33.04 ± 3.32	33.40 ± 3.11				
HGS Left (kg)	Fasting	32.40 ± 4.32	31.81 ± 4.40	0.163	0.695	0.016	
	Eating	31.86 ± 3.49	31.81 ± 4.93				
Jumping (cm)	Fasting	175.11 ± 20.97	168.66 ± 25.63	1.692	0.230	0.175	
	Eating	167.66 ± 18.94	170.11 ± 18.98				

RPE, Rate of perceived exertion; HGS, Handgrip strength. Note: data expressed as the mean ± standard deviation (SD).

**Table 2:** During exercise HRV and performance time.

	Fasting	Eating	t	*p*	d
Performance Time (min)	12.02 ± 2.4	11.24 ± 2.22	1.911	0.085	0.32
Mean Heart Rate (bpm)	161.00 ± 13.26	160.81 ± 8.43	0.045	0.965	0.01
LF Power (ms^2^)	32,493.79 ± 56,646.62	22,355.43 ± 42,151.57	0.442	0.668	0.20
HF Power (ms^2^)	88,773.22 ± 200,141.76	52,884.66 ± 139,620.94	0.455	0.659	0.20
RMSSD (ms)	82.70 ± 49.52	76.83 ± 56.82	0.277	0.787	0.11

Min, minute; bpm, beats·min−1; LF, low-frequency power; ms^2^, milliseconds squared; HF, high-frequency power; RMSSD, root mean square of the successive differences. Statistical significance set at *p* < 0.05. Note: data expressed as the mean ± standard deviation (SD).

## Data Availability

All the data are presented in the study.
